# The Real Ideal: Misestimation of Body Mass Index

**DOI:** 10.3389/fgwh.2022.756119

**Published:** 2022-05-30

**Authors:** Ellie Aniulis, Ella K. Moeck, Nicole A. Thomas, Gemma Sharp

**Affiliations:** ^1^Department of Psychiatry, Central Clinical School, Monash University, Melbourne, VIC, Australia; ^2^Melbourne School of Psychological Sciences, The University of Melbourne, Melbourne, VIC, Australia; ^3^College of Education, Psychology, and Social Work, Flinders University, Adelaide, SA, Australia

**Keywords:** body dissatisfaction, body image, perception, body weight, self-assessment, appearance, women's health

## Abstract

In Western cultures, the ideal body for women is thin and toned. Idealization of thinness has led many women to desire bodies with an underweight body mass index (BMI). The present study investigated women's knowledge of BMI, particularly relating to their own body ideals, to determine whether women knowingly idealize bodies categorized as “underweight.” In August 2020, one-hundred and forty-seven US women aged 18 to 25 completed two online tasks in a repeated-measures design. First, participants estimated the BMIs of a series of bodies. Then, participants selected representations of their own and ideal bodies from a figure rating scale and estimated the BMIs of their selections. Participants generally mis-estimated the BMI of bodies, but did so to a greater extent when viewing bodies as an extension of their own, i.e., following the figure rating scale task. Further, if participants selected an underweight or overweight ideal body, they were likely to estimate this body was within a “normal” weight BMI range, demonstrating that women who idealize underweight–or overweight–bodies do so unknowingly. These findings suggest misperceptions of women's own ideal body size are often greater than misperceptions of other bodies, potentially driving the tendency to idealize underweight bodies.

## Introduction

Body dissatisfaction is the combination of our thoughts, feelings, and perceptions about our body (1). Body dissatisfaction is highly prevalent and a known precursor to the development of eating disorders (2). Eating disorders impact 7.8% of the global population and have the highest mortality rate of all mental health diagnoses (3, 4). Thus, there is a great need to investigate factors that influence judgements of ideal body size in an effort to address the incidence of body dissatisfaction and eating disorders.

One way of measuring body dissatisfaction is to look at the discrepancy between the body size we have and our desired body size—the ideal (5). Ideal body size for women in Western cultures focuses on a thin and toned physique, which is perpetuated by traditional and social media, peers, and family (6–8). In particular, the perpetuation of the thin ideal by mainstream media has long been criticized for its negative impact on women's body image (5, 9). Underweight models are frequently featured in the media (10, 11), further skewing the perception of ideal body size (12). A thin and toned physique is consistently idealized, despite being unattainable for most women. The current study sought to investigate whether women are aware when they are idealizing underweight bodies, or whether they unknowingly do so due to holding inaccurate perceptions of what a categorically “normal” weight body mass index (BMI, height/weight^2^) looks like.

Social comparison is one driving force behind the negative affect caused by overexposure to the thin ideal (13). According to social comparison theory, women make judgments about their own body in relation to the other bodies they are exposed to, generating either an upward (in which they judge their own body more negatively than the other) or downward (in which they judge their body more positively than the other) comparison. The consistent portrayal of thin ideal images in the media leads to frequent upward comparisons and subsequent body dissatisfaction (14, 15), in contrast to exposure to average-sized fashion models which can promote positive body image (16). The high prevalence of social media use likely further exacerbates this issue. Social media makes it easy for people to quickly edit images, leading users to feel pressure to post “perfect” images of themselves that, in turn, can increase body dissatisfaction (17–20).

Body dissatisfaction and desired weight are often measured using figure rating scales (21). In a figure rating scale task, individuals are presented with an image array of bodies increasing in weight. Individuals then indicate which body in the array is the closest to their own body, and which is the closest to their ideal body. The distance between these two selections is used as a measure of body dissatisfaction, indicating how far a person's own body is from their ideal body (21, 22). The bodies in these scales typically have a known BMI that is used to infer the individual's desired weight range, e.g., whether they are idealizing an underweight body. BMI (kg/m^2^) is used to estimate an ideal weight range for health (23). Using WHO classifications of BMI, people are classed as either “underweight” (BMI < 18.5), “normal weight” (18.5–24.9), “overweight” (25.0–29.9), and “obese” (≥30.0). BMI can sometimes be a suboptimal measure which does not, for example, account for body fat changes that occur due to age, sex, muscle mass, and the distribution of fat, and is also less appropriate for non-Caucasian samples (24, 25). Despite these criticisms, BMI is a widely used, quantifiable, and easily applied health measure, which is why we are using it in the present research. BMI is also important in eating disorder diagnoses, particularly anorexia nervosa, with BMI cut-offs determining diagnosis severity (26).

There is some evidence to suggest women might unknowingly idealize underweight bodies. Using figure rating scales, Aniulis et al. (27) found that the most frequently selected ideal body had a BMI of 19.79, closely followed by an underweight ideal of 18.26. Ahern et al. (28) found that a BMI of 20 was considered the most attractive, while Swami et al. (29), and MacNeill and Best (30), found that an underweight body was most frequently selected. Moussally et al. (31) found that participants were able to classify digital body stimuli into their correct BMI categories approximately two-thirds of the time, with incorrect guesses primarily occurring on the cusp of different BMI categories. Incorrect guesses were particularly prevalent on the cusp of the underweight and normal weight classifications. The authors posited these misclassifications could be due to the impact of Western societies' thinness standards—perpetuated by an overexposure to thin ideal ideas—leading to a distorted perspective of a thin body as a normal weight body.

One factor that affects responses to figure rating scales is the context in which the figures are presented. Aniulis et al. (27) found that selections were influenced by the types of bodies presented in the scale, e.g., an over-representation of smaller bodies led to a lower-BMI ideal body being selected. Bair et al. (32) and Mills et al. (33) found that presenting data relating to peer norm body ideals, or average population BMIs, also influenced figure rating scale responses. Interestingly, BMI estimation is also context dependent. Firstly, BMI estimation is typically more accurate around similar bodies to one's own BMI (34, 35). This phenomenon is known as contraction bias. Contraction bias occurs due to a high level of exposure to one's own body, creating a standard point of comparison for other-body judgements. Resulting from this phenomenon, those with lower BMI typically overestimate the BMI of other bodies, while those with higher BMI typically underestimate (34). Secondly, women tend to misestimate the BMI of their own body to a greater extent than that of other bodies, potentially due to the internalized importance of body size on self-image (34, 36). The importance of context in both body size estimations and figure rating scales raises the possibility that women are also more greatly misestimating the size of a body not only when it is presented as a representation of their own body, but also when it is presented in the context of their body ideals.

The present study advances the knowledge of BMI estimation in women, by investigating whether enlarged own-body misestimation also extends to one's own body ideals–i.e., whether there is a greater misestimation for other self-relevant bodies. It was anticipated that participants would generally over-estimate the BMIs of the presented bodies. It was also predicted that participants would overestimate the BMI of the same body to a greater extent when it was selected as a representation of their own body, i.e., from a figure rating scale, compared to when it was presented during the general estimation task, in which participants estimated the BMI of a series of bodies unrelated to their own. These two measurements allow investigation of estimation differences when viewing a body *objectively* as a figure in a general estimation task, vs. viewing a body *subjectively* as an extension of one's own internalized ideals or own body perceptions in a figure rating scale task. In addition, it was expected that participants who selected an underweight ideal body would overestimate the BMI of this body, placing it in the normal weight range. The data from the figure rating scales was used to compare the difference between the level of body dissatisfaction indicated by participants *actual* selections, vs. their *perceived* selections.

## Method

This experiment was approved by the Monash University Human Research Ethics Committee and was performed in accordance with the 1964 Declaration of Helsinki ethical standards.

### Participants

Participants were 147 women aged between 18 and 25 (*M* = 23.23, *SD* = 1.79) from the United States. This sample size was based on an a-priori G^*^Power analysis for a moderate effect size for own and other BMI misperception in a 2 x 2 repeated measures ANOVA (80% power, alpha = 0.05, *f* = 0.30) (37). We chose this effect size based on Tovee et al. (35) who found a medium-sized effect for misperceptions between own and other bodies. In addition to providing adequate power, this sample size is large enough to generate ideal body sizes across the BMI spectrum (27). Of the 147 participants, 107 (72.8%) identified as white/Caucasian, 13 (8.8%) identified as black/African American, 12 (8.2%) identified as Hispanic, 10 (6.8%) identified as Asian, 1 (0.7%) identified as Native American, and 4 (2.7%) were of unknown ethnicity (either due to not responding to the question, or entering their nationality as their ethnicity, e.g., “American”). Participants had a mean self-reported BMI of 25.56 (6.87) placing them in the slightly overweight BMI category, which is representative of the US population (38). Overall, 11 participants (7.6%) were classed as underweight, 73 (50.3%) were classed as normal weight, 26 (17.9%) were classed as overweight, and 35 (24.2%) were classed as obese.

Participants were recruited from Mechanical Turk (MTurk) via CloudResearch (39). MTurk is a viable medium for collecting data on body image related perceptions and attitudes, and produces results comparable to those found in a lab setting (40, 41). Samples from MTurk are typically more diverse and representative than an undergraduate sample, however workers tend to have both a higher education level and lower socioeconomic status than the general population (42, 43). We took several steps to ensure high quality data and prevent bots and server farmer responses (i.e., participants who use commercial data centers to hide their true IP address and complete multiple surveys for financial gain). Participants needed to have a HIT approval rate of 95 or higher, and have at least 1000 HITs approved. Through CloudResearch, survey completion was also restricted to US women only. To enter the survey, participants had to complete a Captcha, and had to correctly answer a simple arithmetic question (e.g., 3 + 4 = ?) presented as an image (which is harder for bots to detect than text). Participants were also presented with multiple choice questions to confirm that they met the survey requirements (women between the ages of 18–25). Qualtrics Expert Review features were also employed to detect possible repeat responders, which can indicate server farmers are completing the survey. Any participants flagged using these features were removed from the survey. Responses were also screened for any abnormally fast responses, of which there were none. Additionally, after these screenings, any participant who consistently made the same ratings, despite obvious changes in body size, was excluded due to likely inattentive survey completion (*n* = 2). This process left a final sample of 145 participants.

### Materials

#### Body Stimuli

Stimuli were selected from Moussally et al. (31) database of digitally-created bodies. These bodies were created using 3D modeling software, and are uniform in height, skin-tone, and attire, and differ only on the spectrum of weight. BMI of these bodies was calculated by estimating the height and weight of the bodies [see Moussally et al. (31) for further details]. For this study, 20 bodies were selected that ranged from 13.19 to 39.1 BMI, with each body increasing by approximately 0.5–2 BMI points.

#### Figure Rating Scales

Figure rating scales were created by presenting the 20 selected bodies in an array, from smallest to largest. Participants were presented with this same scale twice. First, they were asked to select which body on the scale was the closest to their *own* body, then which body was the closest to their *ideal* body. Scores were calculated by subtracting the selected own from the selected ideal body size. A 20-item figure rating scale is longer than those typically used in body image research and can allow more nuance to be detected in ideal/own body discrepancies (21, 31).

#### Visual Analog Scales

Four VAS scales were used to measure state body dissatisfaction, or the level of body dissatisfaction participants were currently feeling after completing the task. VAS scales are frequently used to measure state body dissatisfaction, as they are able to detect greater nuance in state body dissatisfaction levels (44, 45). VAS scales show high test-retest reliability (46), and correlate highly with trait body dissatisfaction measures (44). The VAS scales used in this study measured current feelings of fatness, strength, body dissatisfaction, and appearance dissatisfaction (44, 45). In this instance, to respond to the VAS scale, participants moved a slider along a continuous scale. Scores on each scale could range from 0 to 100, with higher scores indicating higher levels of state body dissatisfaction. These scales were used to calculate a composite measure of body dissatisfaction by averaging scores across the four items, with scores for “strength” reverse coded. The VAS scales showed good reliability in the current study (α = 0.85).

#### Body Shape Questionnaire

The BSQ-M [(47), (27), manuscript in preparation] assessed trait body dissatisfaction. Participants respond to 34 items regarding their thoughts and feelings about their bodies over the past 4 weeks, and rate the frequency of those thoughts on a Likert scale from 1 (Never) to 6 (Always). Scores of the BSQ-M range from 34 to 204, with higher scores indicating greater body dissatisfaction. The BSQ-M showed good reliability (α = 0.98) and the BSQ correlates with scores on figure rating scales (22).

### Procedure

After providing consent, participants were given written information regarding what BMI is, how it is calculated, and cut-off scores for entering the different BMI categories (e.g., underweight, obese) according to WHO. Participants then completed the general estimation task in which they viewed the 20 digital bodies, one at a time, and estimated what they predicted the BMI of each body to be on a sliding scale anchored at 13 on the left and 47 on the right. The slider began in the center of the scale. Participants viewed each body twice, in a random order, and made a rating each time. Following these singular ratings (called the “general estimation task”), participants were presented with the figure rating scale. Participants completed the figure rating scale after the general estimation task to avoid increasing the salience of one's own body prior to completing the general estimation task. To complete the figure rating scale, participants first selected their own body on the scale. The body they selected was then presented on its own, and participants were asked to rate the BMI of the body on the same sliding scale they used in the general estimation task to create their own body BMI estimation. The same process was then performed for the ideal body, generating an ideal body BMI estimation. Participants then completed the VAS scales, the BSQ, and provided their height and weight measurements [used to calculate BMI (48)]. At the conclusion of the study, participants were also asked if they already had knowledge of their own BMI while completing the study, either indicating “yes,” “no,” or “I had some idea, but I wasn't completely sure.” The study took approximately 10–15 min and participants were paid US$1.20. Data were collected in August of 2020.

## Results

### Preliminary Analyses

This study used a repeated-measures design. To investigate the potential influence of prior knowledge of own BMI, an accuracy score was calculated to determine the difference between participants' actual BMI (as calculated by the self-reported height and weight measurements), and their estimated own BMI from the sliding scale measure. Participants' estimated BMI scores were subtracted from their actual BMI scores. In this instance, scores of 0 indicated that participants were entirely accurate at estimating their BMI on the sliding scale, while positive and negative scores indicate over- and under-estimation on the sliding scale, respectively. A one-way ANOVA was performed investigating accuracy differences between those who had indicated they either had full knowledge (*n* = 16, 11.0%), some knowledge (*n* = 80, 55.2%), or no knowledge (*n* = 49, 33.8%) of their BMI. Participants typically overestimated their BMI to a small amount using the slider (*M* = 0.14, *SD* = 3.67), though this was not significantly different from 0 (*t* (144) = 0.46, *p* = 0.647, *d* = 0.04). Interestingly, there was no significant difference in accuracy between the three groups, *F* (2, 142) = 2.505, *p* = 0.085, $\eta_{\text{p}}^{2}$ = 0.034. Thus, all participants were analyzed together.

### Ideal Body Selections

**Figure 1:**
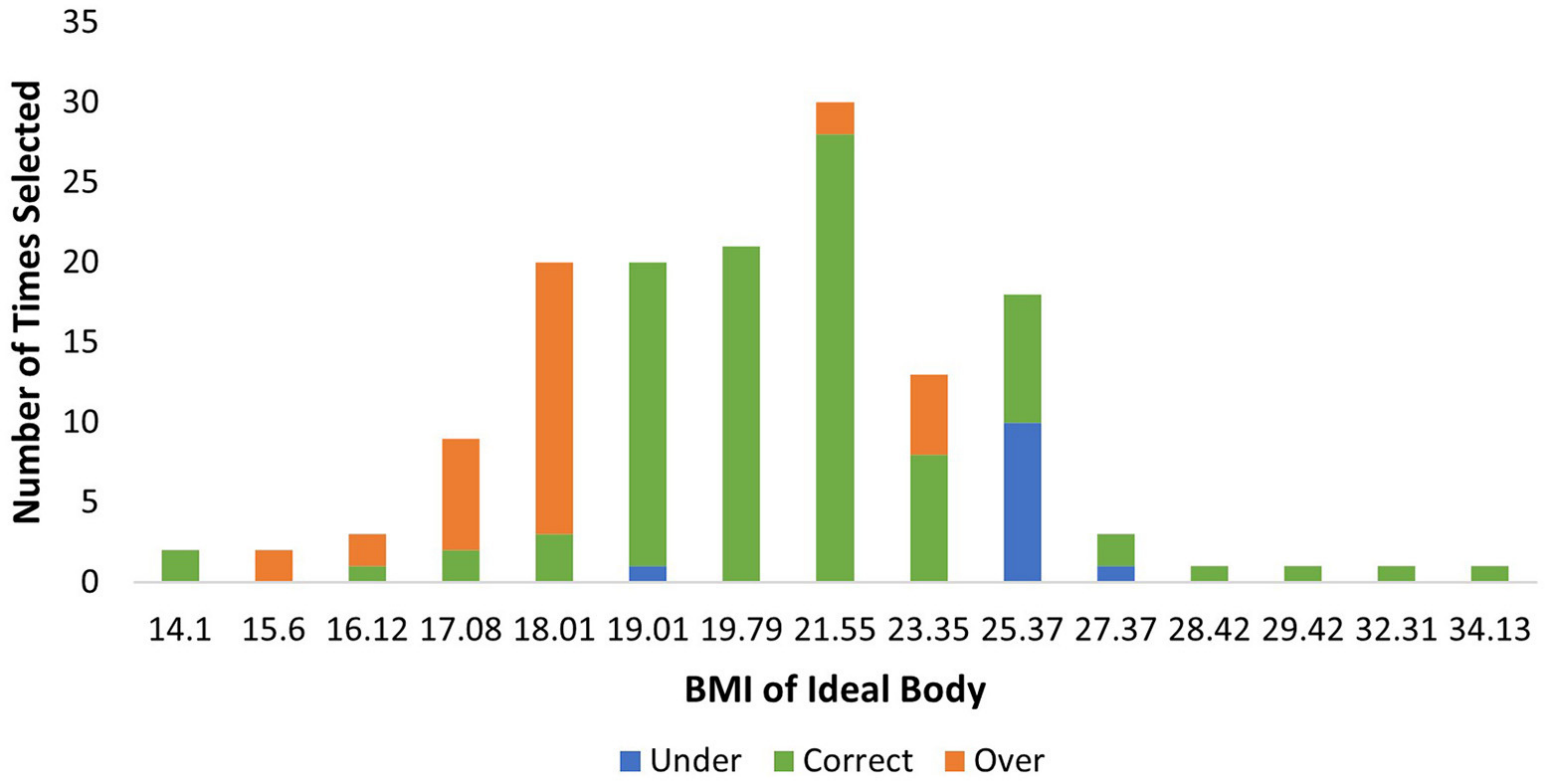
Distribution of ideal body selection frequencies by BMI, with classification accuracy indicated.

It was hypothesized that participants who selected an ideal body would overestimate the BMI of this body, placing it in the normal weight range. Of the 145 participants, 36 (24.8%) selected an ideal body that was underweight [*n* = 2 (5.6%) selected a body with a BMI of 14.1, *n* = 2 (5.6%) selected 15.06, *n* = 3 (8.3%) selected 16.12, *n* = 9 (25%) selected 17.08, and *n* = 20 (55.5%) selected 18.01]. Of these bodies, BMI was typically overestimated by 2.11 points (*SD* = 2.00), with this overestimation being significantly >0 (*t* (35) = 6.34, *p* < 0.001). Only 8 (22.2%) of these participants correctly estimated that the BMI of their selected ideal body was underweight, while the remaining 28 (77.8%) participants estimated their ideal selections to be in the normal weight range (see Figure 1).

Twenty-five participants (17.2%) selected an overweight or obese body as their ideal body [*n* = 18 (72%) selected 25.37, *n* = 3 (12%) selected 27.37, *n* = 1 (4%) selected 28.42, *n* = 1 (4%) selected 29.42, *n* = 1 (4%) selected 32.31, and *n* = 1 (4%) selected 34.13]. Of these bodies, the BMI was typically underestimated by 0.16 (*SD* = 2.77) points, though this underestimation did not significantly differ from 0 (*t* (24) = −0.29, *p* = 0.772). Fourteen participants (56.0%) accurately estimated the BMI to be within the overweight to obese weight range, while 11 (44.0%) estimated the bodies to be within the normal weight range.

The remaining 84 participants selected a normal weight body as their ideal body [*n* = 20 (23.8%) selected 19.01, *n* = 21 (25.0%) selected 19.79, *n* = 30 (35.7%) selected 21.55, and *n* = 13 (15.5%) selected 23.35]. Of these bodies, BMI was typically overestimated by 1.10 points (*SD* = 1.10), with this overestimation being significantly >0 (*t* (83) = 5.60, *p* < 0.001). The majority of participants (*n* = 76, 90.5%) accurately estimated these bodies to be within the normal weight range, while 1 (1.2%) underestimated into the underweight range, and 7 (8.3%) overestimated into the overweight to obese weight range.

To further investigate the accuracy of classifications of ideal body selections, a contingency table analysis was performed. Ideal body estimations were categorized as either accurately classified, classified lower (e.g., a normal weight body being estimated as underweight), or classified higher (e.g., a normal weight body being estimated as overweight). A 4(BMI classification: underweight, normal weight, overweight, obese) x 3(classification accuracy: classified lower, accurately classified, classified higher) chi-square analysis was performed. There was a significant relationship, χ^2^ (6, *N* = 144) = 121.139, *p* < 0.001, φ_*c*_ = 0.649, indicating a difference between the groups. In accordance with Beasley and Schumacker (49), adjusted residuals were used to generate *p* values for each cell. A Bonferroni-adjusted *p* value of 0.004 (for 12 comparisons) was used to determine significance. Participants had a lower-than-expected number of accurate classifications for both those that selected underweight (*p* < 0.001) and overweight (*p* < 0.001) ideals, while there was a higher-than-expected number of accurate classifications for those selecting normal weight ideals (*p* < 0.001). Participants were also significantly more likely to classify underweight ideals higher, and overweight ideals lower, into the normal weight classification (*p*'s < 0.001). Normal weight ideal bodies had a lower-than-expected number of both lower (*p* = 0.001) and higher classifications (*p* < 0.001). These results suggest that participants were quite inaccurate at estimating the BMI of ideals that fell beyond the normal weight category, indicating they unknowingly idealized both underweight and overweight bodies (see Figure 1).

### Estimation Patterns for Own, Ideal, and Other Bodies

We next investigated whether participants showed a *greater* overestimation for both own and ideal bodies when making size estimates after a selection on a figure rating scale (a self-judgement), than when estimating the same body during the general estimation task (an other-judgement). We first calculated the mean of these ratings for each judgment type, which suggested participants typically selected an ideal body that was smaller than their own body (see Table 1). We then formally tested this possibility using a misperception score, where we subtracted the actual BMI of the image from the estimated BMI of the image. Negative scores indicate BMI underestimation, and positive scores indicate overestimation, whereas 0 indicates accurate estimation. A 2 (Body Type: Own, Ideal) x 2 (Judgement Type: Self, Other) repeated measures ANOVA was performed on BMI misperception scores (see Table 2). Comparisons were made between the bodies selected as representations of their own and ideal bodies in the figure rating scale, and the equivalent bodies per participant in the general estimation task.

**Table 1:** Mean BMI estimations for both own and ideal bodies, made during the figure rating scale (self) or the general estimation task (other).

		**Body type**		
		**Own**	**Ideal**	**Overall**
Judgment type	Self	25.70 (5.50)	22.03 (3.35)	23.87 (3.85)
	Other	25.28 (5.29)	21.71 (3.72)	23.49 (3.98)
	Overall	25.49 (5.24)	21.87 (3.37)	

**Table 2:** Mean BMI misperception scores for both own and ideal bodies, made during the figure rating scale (self) or the general estimation task (other).

**Judgment type^b^**	**Body type** ^**a**^		
	**Own *M* (*SD*)**	**Ideal** ***M* (*SD*)**	**Overall *M* (*SD*)**
Self	0.84 (2.61)	1.14 (2.16)	0.99 (1.98)
Other	0.41 (2.57)	0.81 (2.38)^c^	0.61 (2.25)
Overall	0.62 (2.23)	0.98 (1.98)	

a *Main effect of body type: F (1, 144) = 4.62, p = 0.033, $\eta_{p}^{2}$ = 0.033*.

b *Main effect of judgment type: F (1, 144) = 4.97, p = 0.027, $\eta_{p}^{2}$ = 0.027*.

c *Interaction: F (1, 144) = 0.21, p = 0.647, $\eta_{p}^{2}$ = 0.001*.

There was a small but significant main effect of body type, indicating that overall, BMI misperception was higher for ideal than own bodies (*F* (1, 144) = 4.62, *p* = 0.033, $\eta_{\text{p}}^{2}$ = 0.031). There was also a small but significant main effect of judgement type (*F* (1, 144) = 4.96, *p* = 0.027, $\eta_{\text{p}}^{2}$ = 0.033). In line with predictions, estimations were typically higher when judging the bodies following the figure rating scale (self judgments), than in the general estimation task (other judgments). In other words, participants demonstrated greater overestimation patterns when the bodies were associated with their own body size and goals, than when they were presented as standalone bodies without connection to the participant. Though significant, the effect size here was small, representing a difference of 0.3 BMI points between the two judgment types. The interaction effect was not significant (*F* (1, 144) = 0.21, *p* = 0.647, $\eta_{\text{p}}^{2}$ = 0.001), indicating that overestimation patterns were similar for own and ideal bodies (see Table 2).

### Actual vs. Estimated Scores on the Figure Rating Scale

Figure rating scales are typically used as a measure of body dissatisfaction. In a figure rating scale, a participant is presented with a line of figures increasing in size and makes two selections: the body which they perceive as the closest representing their own body, and the body that they consider their ideal body. Figure rating scales provide a measure of body dissatisfaction by demonstrating the distance between one's own body and one's ideal body. To investigate the difference between the level of body dissatisfaction indicated by participants actual selections on the figure rating scale, vs. their perceived selections, a 2 (Body Type: Own, Ideal) x 2 (Score Type: Actual, Perceived) repeated measures ANOVA was performed. Comparisons were made between the actual BMI of the image and the BMI estimation participants made following their selections.

There was a main effect of body type, indicating that BMI for own bodies was typically greater than for ideal bodies, regardless of score type. There was also a main effect of score type, which indicated that participants estimations of the BMI of the image were higher than the actual BMI of the image. This finding fits with women typically overestimating the BMI of bodies. There was no significant interaction (see Table 3).

**Table 3:** Mean figure rating scale scores for own and ideal bodies, from the actual BMI of the selections on the scale and the perceived BMI of the selections.

	**Rating scale scores** ^**b**^	
	**Actual (from scale) M (SD)**	**Perceived (from estimate)** **M (SD)**
Own body	24.87 (5.61)	25.70 (5.50)
Ideal body^a^	20.89 (3.37)	22.03 (3.35)^c^
Difference score	−3.97 (4.78)	−3.67 (4.88)^d^

a *Main effect of body type: F(1, 144) = 98.657, p < 0.001, $\eta_{\text{p}}^{2}$ = 0.407*.

b *Main effect of score type: F(1, 144) = 35.953, p < 0.001, $\eta_{\text{p}}^{2}$ = 0.200*.

c *Interaction of body type by score type: F(1, 144) = 1.783, p = 0.184, $\eta_{\text{p}}^{2}$ = 0.012*.

d *Difference score: t(144) = 1.335, p = 0.184, d = 0.111*.

Scores on the figure rating scale were calculated by subtracting the BMI of the body selected as one's own body from the BMI of the body selected as one's ideal body. Thus, a negative score indicated that a participant desired a body smaller than their own, while a positive score indicated that a participant desired a body larger than their own. In this instance, two scores were calculated: one using the *actual* BMI of the selections, and one using the *perceived* BMI of the selections (from the estimations made by the participants). To determine if overestimation affected difference scores on figure rating scales, a paired samples *t*-test was performed to investigate the difference scores between own and ideal BMI for both the actual image selections and the estimation of the selections. There was no significant difference between the difference scores, suggesting that actual and perceived scores on the figure rating scale demonstrated equivalent scale scores. This indicated that, though the bodies in the figure rating scale were typically overestimated, the level of body dissatisfaction calculated from the scale remained consistent (see Table 3).

As figure rating scale scores are known to correlate with other body dissatisfaction measures, both actual and perceived scores on the figure rating scale were correlated with state (measured by the VAS) and trait (measured by the BSQ-M) measures of body dissatisfaction. We used partial correlations, controlling for participant BMI, to prevent body size from confounding the relationship between these variables. Both actual and perceived scores on the figure rating scale showed moderate negative correlations with both state and trait body dissatisfaction (see Table 4). As a negative score on the figure rating scale indicates that one's ideal is smaller than their own body, this negative correlation suggests that greater body dissatisfaction coincided with a desire for a body smaller than one's own.

**Table 4:** Correlations (r) between body dissatisfaction scores and figure rating scale scores, controlling for participant BMI.

	**Figure rating scale scores**	
	**Actual (from scale)**	**Perceived (from estimations)**
State Body Dissatisfaction	−0.268^**^	−0.418^***^
BSQ	−0.204^*^	−0.308^***^

*** *Indicates a p value < 0.001*.

** *Indicates a p value < 0.01*.

* *Indicates a p values < 0.05*.

## Discussion

This study investigated misestimations of own and ideal body sizes, and how these misperceptions may impact figure rating scale scores. Though mean misperception scores were positive, indicating an overall tendency to overestimate body size, results demonstrated that this tendency to overestimate was greatest for smaller bodies, before decreasing to a small but not significant tendency to *under*estimate the BMI of bodies, as they got larger. This tendency to mis-estimate body size provided some support for the first hypothesis. Additionally, a small but significant main effect of judgment type indicated that participants were more likely to mis-estimate bodies to a greater extent when they were representing their own body or their internalized ideals (i.e., in the figure rating scale task) than when they were estimating the size of self-*irrelevant* bodies (i.e., in the general estimation task). This finding provides some support for the second hypothesis. Misinterpretations of body size could potentially contribute to idealization of bodies outside of a normal BMI range. In particular, idealization of thinness, such as a desire for an underweight body, is a known risk factor for eating disorders (50).

We found a tendency for participants to overestimate their own body size, which decreased as participants BMI increased. This finding is in line with results from both Cornelissen et al. (34) and Thaler et al. (36). An underestimation of overweight and obese body sizes is also in line with results from Oldham and Robinson (51) and Robinson et al. (52). Prior work has also demonstrated that misperceptions of own body size occur to a greater extent than misperceptions of the body sizes of others (34). This study aimed to see if this enlarged misperception extended to other self-relevant bodies–in this case, the ideal body. To our knowledge our findings are the first indication of an enlarged misperception of ideal body size in the context of the self-vs.-another. However, the size of the effect between self vs. other misperceptions was small, representing a difference of ~0.3 BMI points—future research should investigate the robustness of this effect. Results suggested that misperceptions of the ideal were not consistent across BMI classifications. Therefore, future investigation of this effect could use groups at the outer ends of the BMI scale–those who are underweight or overweight–to see if self-relevant bodies are more greatly misperceived in these groups.

In line with our third hypothesis, participants who selected an underweight body as an ideal were likely to miscategorise this body as being in the normal weight range. Interestingly, and outside the scope of our hypotheses, the reverse was seen for those who indicated an overweight body as their ideal, with these participants instead frequently underestimating the BMI of their desired body, also placing it in the normal weight range. Moussally et al. (31), using the same stimuli as in the present study, also found that miscategorisations of BMI were common, particularly for bodies on the cusp of the classifications. These results are also in line with the contraction bias seen by Cornelissen et al. (34) and others (51, 52), in which participants with low BMI generally overestimated their body size, and those with high BMI underestimated their body size. Additionally, Bair et al. (32) and Mills et al. (33) indicated that ideal body size trended toward purported ideal peer and population norms, respectively. Furthermore, Robinson and Kersbergen (53) found that perceptions of one's own weight status (e.g., being overweight) were influenced by the weight of a perceived “average” person.

As terminology for BMI classifications presents an indication of a “normal” weight, it is possible that participants urged to fit their own desires into this perception of normality. Indeed, BMI is criticized for promoting the use of stigmatizing terms such as “obese” (54), which could have encouraged participants both away from selecting these ideals, and toward skewing their own ideals into the normal weight category. Future research should investigate how ideal body sizes might be categorized if these indications of normality were manipulated, e.g., by using generic terms to refer to the weight classifications (Category A, Category B, etc.). Overall, these results suggest that a drive toward a normal weight range–which, in the context of BMI, would be considered a “healthy” body–is common, but women's perception of what this body looks like is skewed and misunderstood. This skewed perception likely stems from a consistent over-exposure to and glorification of thin ideal images, sociocultural pressure for women to have and maintain and thin physique, and the over-emphasis on the importance of women's appearance (19, 55, 56). This idea is consistent with objectification theory (55), which posits that cultural objectification of women's bodies leads to an internalization of the expectations that others have for their bodies. This internalization amplifies body monitoring behaviors, leading to an increased risk of eating disorders and other mental health issues.

The results of the present study have practical implications for figure rating scales. They suggest that overall difference scores on a figure rating scale are not impacted by participant misestimations of body size because the BMI of own and ideal bodies were overestimated to a similar degree. Therefore, the overall difference score between the two values–used to estimate body dissatisfaction–remained statistically similar. However, due to frequent miscategorisations, it appears that body selections on figure rating scale should not be extrapolated to indicate a participant's desired weight category, or an exact representation of their desired ideal weight. These data suggest that future endeavors from this research are twofold: First, providing education regarding what the different classifications of BMI look like, and second, perpetuating that no particular body represents a singular ideal, given BMI does not always correlate with physical health (57), or normality. Moving beyond the thin ideal and into an era of body positivity and body diversity is essential to inform women's choices of ideal body size. Retreating from a current social norm in which only women who conform to narrow sociocultural standards of beauty are celebrated, and toward celebration of bodies of all types, shapes, and abilities–in line with the body positivity movement (58)–will help broaden ideas of what is “ideal” and potentially help protect against eating disorder risk (59, 60).

Additionally, the figure rating scale provides iterations of the same body, changing only on the dimension of weight. However, body ideals also revolve around dimensions of muscularity and weight distribution, e.g., a desire for a smaller waist but larger hips. Dissatisfactions of this kind cannot be inferred by figure rating scale selections. A further exploration of ideal body size perceptions and misperceptions, using similar methodology to Matsangigou et al. (61) in which participants manipulated a single body on multiple dimensions, would more comprehensively inform ideal body size overestimations.

Indeed, this study is limited by the stimuli used, which are removed from real bodies. The digital body database (31) provides a highly controlled set of stimuli; the bodies have a known BMI and, other than differing on weight, are identical. These bodies are typical for figure rating scale (31, 62), which made them suitable for our aim of investigating the impact of body size perceptions on figure rating scale. However, our investigation of own and ideal body sizes required participants to see the stimuli as a representation of their own body. Though these figures have previously been rated as plausible representations of bodies (31), due to this methodological limitation we are unable to determine if participants successfully embodied the stimuli. This lack of embodiment could be a partial driver of the small effect size and borderline significant *p*-value found between estimations for self and other bodies.

Our sample was also limited by comprising primarily Caucasian women from a young age bracket. People of other genders and ethnicities, and older women, also experience body dissatisfaction (63–66). Exploring ideal body size estimations in these groups would help create a clearer picture of how BMI misperceptions may alter depending on demographics. Additionally, our participants were not screened for prior history of eating disorders, which could have impacted performance on the task, as BMI misperceptions are typically greater in those experiencing eating disorders (34). Though participants were given a brief overview of what BMI is and how it is calculated at the commencement of the experiment, and were asked about their knowledge of their own BMI, participants were not screened for their knowledge of BMI in general. Participants without this general knowledge of BMI may have struggled to fully conceptualize how it translates to different body shapes.

Finally, an important limitation of this study is that the figure rating scale task was completed at the conclusion of the study for all participants, leaving open the possibility that misperceptions on this task were influenced by the prior completion of the general estimation task. We used a sequential order to avoid increasing the salience of participants' own bodies prior to completing the general estimation task. However, future studies should present these tasks in a counterbalanced order. In addition, BMI scores in the general estimation task were generated from an average score of two ratings, while the figure rating scale score used a single rating, with the average score potentially leading to more precise estimates in the general estimation task. At present, the potential for order effects, combined with the small effect size, suggests the difference in misperception between the two tasks should be interpreted with some caution. However, these results do provide a platform for further investigation into the magnitude of ideal body size misperception.

Overall, the results of the present study suggest that ideal body size is an important representation of the self, which is often perceived inaccurately. Though it was most common to pick an ideal body size that was in the normal weight range, some participants also picked ideal bodies that were underweight or overweight, and estimated that these bodies were also representations of the normal weight range. A priority of future research should be to investigate whether misperceptions of ideal body size are contributing to the pursuit of unsustainable or unhealthy bodies in young women.

## Data Availability Statement

The raw data supporting the conclusions of this article will be made available by the authors, without undue reservation.

## Ethics Statement

The studies involving human participants were reviewed and approved by Monash University Human Research Ethics Committee. Written informed consent for participation was not required for this study in accordance with the national legislation and the institutional requirements.

## Author Contributions

EA, EM, NT, and GS contributed to study conception and design. EA conducted data collection, statistical analysis, and manuscript preparation. EM and GS contributed to manuscript revision. GS and NT contributed to supervision. All authors contributed to the article and approved the submitted version.

## Funding

Society for Applied Research in Memory and Cognition (SARMAC) Student Research Grant and Australian Government Research Training Program (RTP) Scholarship.

## Conflict of Interest

The authors declare that the research was conducted in the absence of any commercial or financial relationships that could be construed as a potential conflict of interest.

## Publisher's Note

All claims expressed in this article are solely those of the authors and do not necessarily represent those of their affiliated organizations, or those of the publisher, the editors and the reviewers. Any product that may be evaluated in this article, or claim that may be made by its manufacturer, is not guaranteed or endorsed by the publisher.
